# Foley catheter technique for the extraction of coins lodged in the upper esophagus of children

**DOI:** 10.1186/s12887-023-04328-z

**Published:** 2023-11-30

**Authors:** Basak Erginel, Meltem Kaba, Cetin Ali Karadag, Abdullah Yildiz, Mesut Demir, Nihat Sever

**Affiliations:** 1 Istanbul Medical Faculty, Department of Pediatric Surgery, Istanbul University, Istanbul, Turkey; 2 Department of Pediatric Surgery, Sarıyer Hamidiye Etfal Training and Research Hospital, Istanbul, Turkey

**Keywords:** Foley balloon catheter, Esophageal foreign body, Coin, Children

## Abstract

**Introduction:**

Coins are the most commonly ingested foreign bodies in children. They usually become lodged in the upper oesophagus and should be managed immediately. The aim of the present study was to evaluate the characteristics and outcomes of patients with coins lodged in the upper oesophagus, who underwent coin removal using a silicone Foley balloon catheter without fluoroscopy or anaesthesia and evaluate the safety of the procedure.

**Materials and methods:**

Patients who were admitted from January 2007 to December 2022 for coins lodged in the oesophagus and extracted with silicone Foley balloon catheter without anestehesia were evaluated retrospectively. We focused on the patient characteristics and clinical presentations, and the treatment safety, efficacy, and outcomes.

**Results:**

773 patients (416 male, 357 female), with a mean age of 3.5 years (range 6 months to 16 years), who ingested coin and extracted with Foley catether is included. The majority of patients (*n* = 728, 94.17%) were successfully managed by silicone Foley balloon catheter extraction. Our overall success was 94.17%, with 88.30% of coins retrieved and 5.9% pushed into the stomach. Patients who were successfully treated with Foley catheter were discharged on the same day except for 7 (0.90%) who had minimal bleeding. Only 45 (5.82%) patients required oesophagoscopy in the operating room and these patients were kept overnight for clinical follow-up, without any further interventions.

**Conclusion:**

A Foley balloon catheter can be used to safely and effectively remove coins that are lodged in the upper oesophagus avoiding the risk of general anesthesia.

## Introduction

Foreign body ingestion most commonly occurs during the toddler period of childhood because of the behavior, physiology, and anatomical characteristics of children at that age [1]. Coins are the most frequently ingested foreign body, and the ingested coins usually become lodged within the esophagus, most commonly in the upper and middle Sections [2–4].

Symptoms of the presence of esophageal coins are nonspecific, including choking, respiratory distress, wheezing, dysphagia, drooling, and chest and abdominal pain. The clinical findings depend on the characteristics of the foreign body ingestion, such as the degree of obstruction, the site where the foreign body is lodged, and the length of time elapsed between ingestion and evaluation.

A coin lodged in the esophagus should be considered a clinical emergency [5]. The aims of treatment are to prevent tissue damage and to decrease the risk of complications. It is important to diagnose the presence of and extract the coin from the esophagus as early as possible. The most common approach is extraction of the coin by rigid esophagoscopy under general anesthesia or, more recently, by balloon extraction under fluoroscopic guidance [6, 7]. However, one of these two methods requires general anesthesia, and the other requires additional exposure to radiation [8, 9].

The aim of this study was to evaluate our patients who have undergone extraction of coins lodged in the esophagus retrospectively and the safety of this procedure.

## Materials and methods

Data were retrospectively collected from the medical records of 773 patients (aged 6 months to 16 years, mean age 3.5 years) who were admitted from January 2007 to December 2022 for a coin lodged in the upper esophagus and who underwent silicone Foley balloon catheter extraction after informed consent had been obtained from the parents. This retrospective study was approved by the Local Ethics Committee (Sariyer Hamidiye Etfal Education and Research Hospital- Clinical Research Ethics Committee- 2023).The data included characteristics of the enrolled children, referral time, and treatment outcomes.

Exclusion criteria for the Foley catheter extraction procedure included ingestion of a caustic substance and ingestion of other items (batteries, etc.). Inclusion criteria were imaging evidence of the presence of a round coin lodged in the upper esophagus, witnessed coin ingestion, coins lodged in the esophagus for < 24 h prior to presentation, absence of respiratory distress or esophageal abnormalities.

**Fig. 1:**
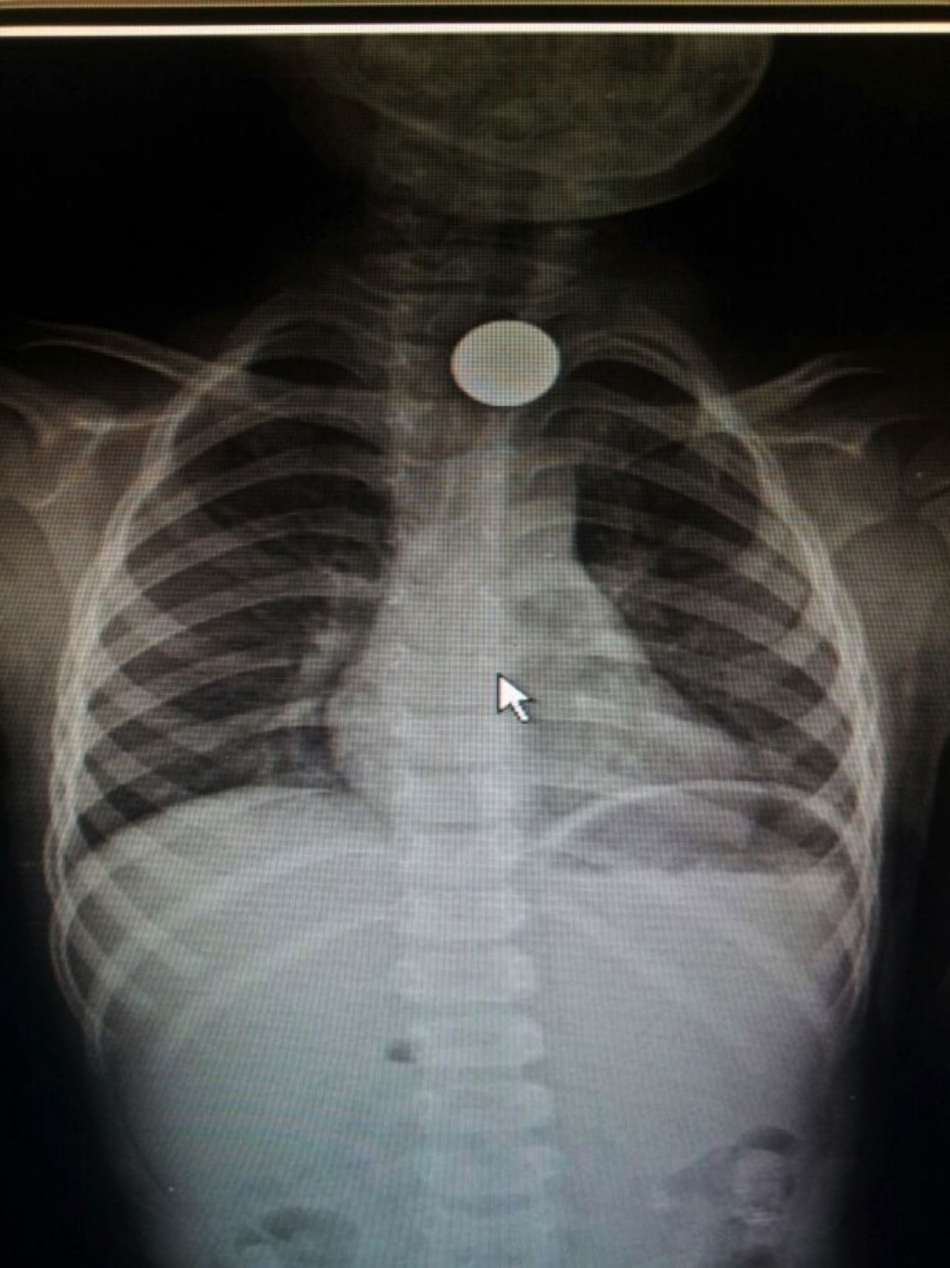
Chest X-ray demonstrating coin in the upper esophagus

Coin extraction was performed in the pediatric surgery department without anesthesia or sedation. Plain chest radiographs were obtained for all patients (Fig. 1). The following equipment was kept ready in the outpatient room: a suitable silicone Foley balloon catheter (12 or 14 F), a 10-mL syringe filled with saline, tongue depressors, laryngoscope, forceps, suction apparatus, oxygen supply, and all other equipment required for emergency intubation. The balloon of the Foley catheter was tested for inflation before the procedure.

**Fig. 2:**
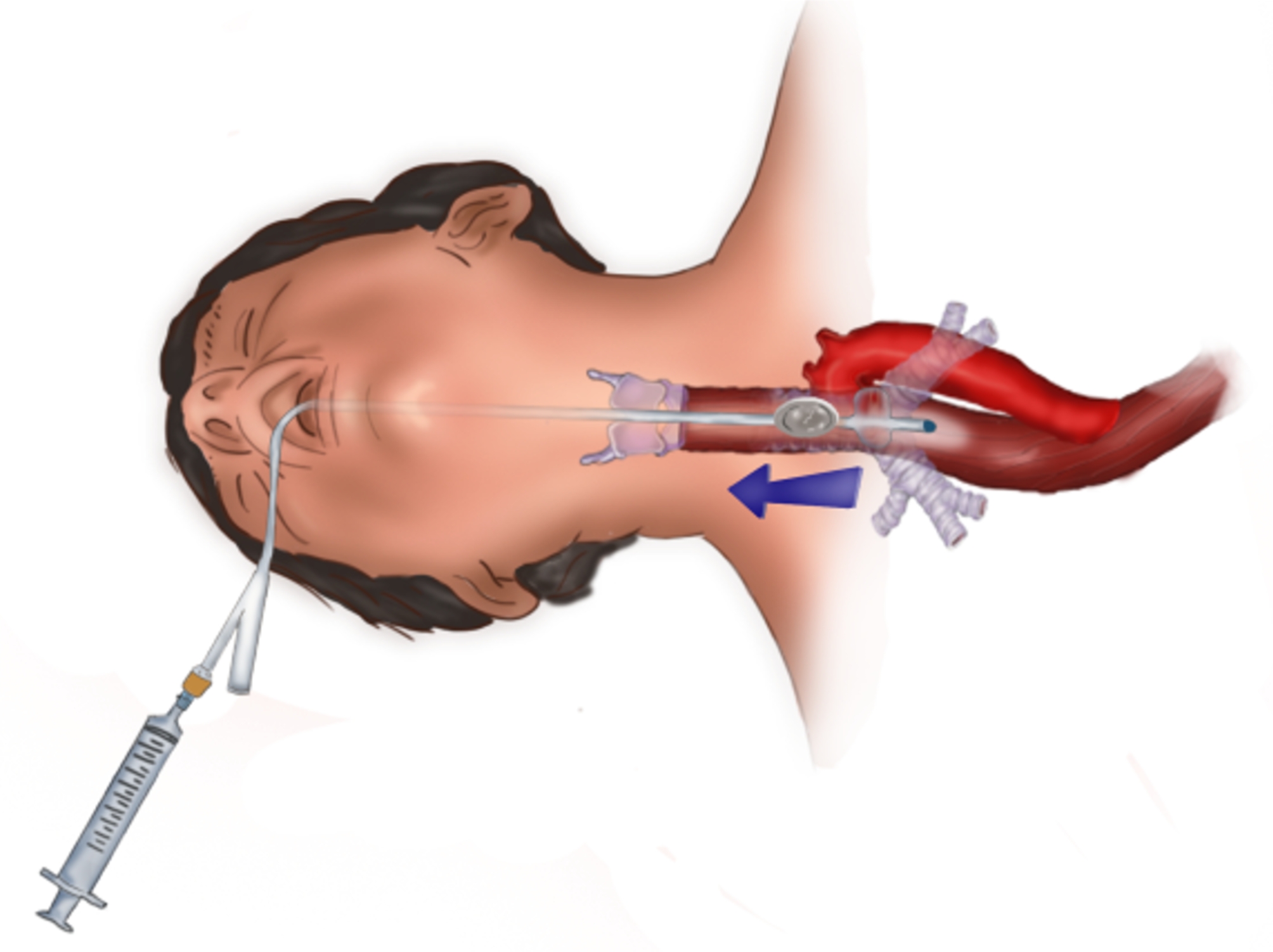
Illustration of the Foley catether technique for the removal of a coin from the upper esophagus

If the patient had a coin lodged in the upper two-thirds of the esophagus, the ingestion had been witnessed, and the time from ingestion to presentation was < 24 h, the patient underwent extraction using a Foley catheter. The catheter was inserted transorally without sedation, allowing the child to cough up the object, thereby assisting the extraction. Tongue depressors were placed intraorally on both sides of the mouth to prevent the child from biting down on the catheter. The lubricated Foley balloon catheter was then passed through and advanced posterior and distal to the lodged coin and inflated with saline (3–5 mL), thus avoiding overdistension. The inflated balloon was then withdrawn carefully (Fig. 2). The patient was turned to the right decubitus position on the side of the table to allow gravity to assist with expectoration. No anesthesia is used for the procedure. When the catheter was completely removed, if the procedure was successful, the coin reached the oral cavity. If the procedure failed, it was repeated up to 3 times. During any of these attempts, the coin may be pushed down into the stomach. After successful extraction or pushing the coin into the stomach, the patients were discharged following careful evaluation, with no further follow-up, and the parents were advised to feed their children.

If the third attempt at Foley catheter extraction proved unsuccessful and the control X-ray reconfirmed the position of the coin as still being in the esophagus, rigid esophagoscopy was performed in the operating room under general anesthesia. The children who underwent rigid esophagoscopy stayed for observation in the hospital for one day.

## Results

A total of 773 children (416 boys and 357 girls) presenting with coin ingestion underwent an extraction procedure using a Foley catheter. The mean age of the patients was 3.52 years (range 6 months to 16 years).

In all cases, the position of the coin was in the upper two-thirds of the esophagus, the total duration of coin impaction was < 24 h, and all ingestions had been witnessed. The time duration from ingestion to presentation was < 6 h in 98% of our cases. All patients underwent radiographic examination for diagnosis and to determine the exact location of the coin in the gastrointestinal tract before intervention.

The most common presenting symptom was drooling, followed by dysphagia, refusal to eat, and vomiting. Unwitnessed ingestions were excluded due to the lack of knowledge of the time elapsed since ingestion or whether the ingested foreign body was a coin.

The removal of impacted coins using a silicone Foley catheter was successful in 728 (94.21%) patients. In 46 of the 773 children (5.9%), the coins were pushed into the stomach, and as no further interventions were needed; we included these cases in our success rate (94.21%). Our overall success rate was 94.17%, with 88.30% of coins retrieved and 5.9% pushed into the stomach. These patients were discharged after careful evaluation, and no further follow-up was required except for those who had minimal bleeding.

In the remaining 45 children (5.82% of cases), all three attempts with Foley balloon catheter extraction were unsuccessful. In these cases, the position of the coin was reconfirmed on radiographs as being in the esophagus, and the coin was then removed by rigid esophagoscopy in the operating room under general anesthesia. The children who underwent rigid esophagoscopy were observed for 1 day after the procedure in the clinic and subsequently discharged.

Minimal bleeding due to mucosal abrasion occurred in 7 (0.90%) patients. No other complications occurred after the extraction. The patients with minimal bleeding were kept overnight for clinical follow-up, without any further intervention. The remaining children for whom the coins were successfully extracted with Foley catheter were sent home on the same day.

## Discussion

Esophageal coin impaction is a common clinical problem occurring among young children and requires an invasive procedure for extraction. The lodged coin is usually located in the upper esophagus. Chewing and swallowing become more coordinated around the age of 5 years, when the incidence of this problem declines. The sex and age distribution, as well as the male predominance, noted in our study population are consistent with those reported in previous studies [2–4].

Removal of coins from the esophagus has always been challenging. The earliest reports indicated that in the seventh century, a piece of sponge tied to a string was used to remove objects from the esophagus [10]. In the more modern era, rigid esophagoscopy has been the gold standard for the removal of objects from the esophagus of children [11]. Although this can be used for all types of esophageal foreign bodies, irrespective of their shape or the material they are made of, balloon extraction has almost completely replaced rigid esophagoscopic removal for the removal of blunt and round objects [12]. In 1966, Bingler reported the first Foley catheter extraction of a blunt object from the esophagus of a pediatric patient [13]. Since Bingley’s report, large series’ of Foley catheter, extraction of coins from the esophagus using fluoroscopy and/or sedation, without associated complications, have been reported [14, 15]. In the present study, extraction was performed using a silicone rather than a rubber Foley catheter. When 12-F catheters are filled with air, the balloon of the silicone catheter inflates symmetrically, becomes more spherical, and distends more firmly than does a rubber catheter. Therefore, in our practice we favor the use of silicone Foley catheters. In a recent series of 819 cases reported by Gasior et al., there were 4 complications: 2 patients had epistaxis; in 1 patient, the balloon was lodged in the nasopharynx, requiring balloon removal with direct laryngoscopy; and another patient had an apnoeic event [16].

Recently, Svetanoff et al. reported on their 30-year experience of fluoroscopic retrieval using Foley catheters in 809 children. They reported their overall success rate as 85.5%, with 76.5 coins retrieved and 9% pushed to the stomach. Our overall success rate was similar at 94.17%, with 88.30% of coins retrieved and 5.9% pushed into the stomach. Our method is similar except we do not use fluoroscopic guidance [17].

The technique of Foley catheter extraction is easy to learn and safe to perform, provided that patient selection is suitable. The main purpose of the procedure is reducing the dose of radiation exposure. Secondly, since anesthesia is not given, the cough reflex is not suppressed, so there is no risk of aspiration, the child easily expels the foriegn body. To ensure the safety and success of the procedure, some basic rules should be followed. This procedure should only be used for blunt and radioopaque coins, specifically excluding button batteries. We avoid performing the procedure in patients undergoing esophageal surgery, those who swallow anything other than money, and those who have waited too long. Although long-term impaction is associated with increased inflammation, edema, and local tissue reaction, which limits the success rates of Foley catheter extraction in relation to the duration of coin impaction, some series have reported safe and successful use of Foley catheters in the extraction of foreign bodies up to 72 h after impaction [18]. We believe that in cases where Foley catheter is used for extraction, the duration of impaction should not exceed 24 h because after this length of time, the success of the procedure decreases, and the esophagus should also be evaluated for any possible pressure damage or perforations. This method should also be avoided if esophageal perforation is suspected. There is no possibility that the coin would escape into the trachea during removal from the esophagus. No such complication is seen in 773 children, because this procedure is performed awake and the child’s cough reflex is not suppressed. Secondly, the diameter difference between the trachea and esophagus is not suitable for aspiration of a coin.

The main limitation of the present study arises from its retrospective and single-center design. However, 15 years of experience with 773 patients is an extensive cohort providing evidence for the safety of this procedure.

## Conclusions

Since no morbidity and mortality were observed in 773 patients, we suggest that the use of Foley catheter without fluoroscopy or anesthesia is a safe method for the removal of coins lodged in the upper esophagus. This approach avoids the need for a long hospital stay or the risks from exposure to radiation and anesthesia. Foley catheters can be used in emergencies such as with the esophageal impaction of coins if endoscopy cannot be performed immediately like rural areas and in patients presenting at midnight in a facility, especially in those without access to endoscopes or emergency services, or in any situation that warrants urgent removal of a foreign object.

## Data Availability

All data analysed during the current study are included in the published article.
